# Determination of Colchicine in Pharmaceutical Formulations, Traditional Extracts, and Ultrasonication-Based Extracts of *Colchicum autumnale* Pleniflorum (L.) Using Regular and Greener HPTLC Approaches: A Comparative Evaluation of Validation Parameters

**DOI:** 10.3390/plants11131767

**Published:** 2022-07-03

**Authors:** Mohammed H. Alqarni, Faiyaz Shakeel, Tariq M. Aljarba, Maged S. Abdel-Kader, Hala H. Zaatout, Sultan Alshehri, Prawez Alam

**Affiliations:** 1 Department of Pharmacognosy, College of Pharmacy, Prince Sattam Bin Abdulaziz University, Al-Kharj 11942, Saudi Arabia; m.alqarni@psau.edu.sa (M.H.A.); t.aljarba@psau.edu.sa (T.M.A.); mpharm101@hotmail.com (M.S.A.-K.); 2 Department of Pharmaceutics, College of Pharmacy, King Saud University, Riyadh 11451, Saudi Arabia; faiyazs@fastmail.fm (F.S.); salshehri1@ksu.edu.sa (S.A.); 3 Department of Pharmacognosy, Faculty of Pharmacy, Alexandria University, Alexandria 21215, Egypt; hala.zatout@alexu.edu.eg

**Keywords:** colchicine, *Colchicum autumnale* Pleniflorum, greener HPTLC, regular HPTLC

## Abstract

In the literature, there is a scarcity of greener analytical approaches for colchicine (CLH) analysis. As a result, efforts were made in this study to develop and validate a greener reversed-phase high-performance thin-layer chromatography (HPTLC) technique for CLH analysis in traditional extracts (TE) and ultrasonication-based extracts (UBE) of commercial Unani formulations, commercial allopathic formulations, and *Colchicum autumnale* Pleniflorum (L.) obtained from Egypt and India. This new technique was compared to the regular normal-phase HPTLC method. The greenness profile of both methods was estimated using the Analytical GREENness (AGREE) approach. In the 100–600 and 25–1200 ng/band ranges, regular and greener HPTLC procedures were linear for CLH analysis, respectively. For CLH analysis, the greener HPTLC method was more sensitive, accurate, precise, and robust than the regular HPTLC method. For CLH analysis in TE and UBE of commercial Unani formulations, commercial allopathic formulations, and *C. autumnale* obtained from Egypt and India, the greener HPTLC method was superior in terms of CLH content compared to the regular HPTLC method. In addition, the UBE procedure was superior to the TE procedure for both methods. The AGREE scores for regular and greener reversed-phase HPTLC methods were found to be 0.46 and 0.75, respectively. The AGREE results showed excellent greener profile of the greener HPTLC method over the regular HPTLC technique. Based on several validation criteria and pharmaceutical assay findings, the greener HPTLC method is regarded as superior to the regular HPTLC approach.

## 1. Introduction

Colchicine (CLH) is an alkaloidal compound that is commonly obtained from *Colchicum autumnale* Pleniflorum (L.) (family: Colchicaceae) [1]. In the traditional system of medicine, CLH is used in the treatment of gout [2]. It has also been found to show anti-inflammatory, antimitotic, and anticancer activity [2,3,4] and is used in the treatment of Mediterranean fever [5]. Recently, CLH has also been investigated in the treatment of SARS-COVID-19 [6,7]. CLH is present in various Unani formulations, allopathic formulations, and Ayurvedic formulations. As a consequence, CLH in pharmaceutical dosage forms and plant extracts must be analyzed qualitatively and quantitatively.

A through literature analysis demonstrated various analytical methods for CLH analysis in marketed formulations, plant extracts, and physiological fluids. For analysis of this compound in its pure form and dosage forms, a spectrophotometry approach has been reported [8]. Various high-performance liquid chromatography (HPLC) assays have been used for its detection in different formulations, medicinal extracts, and microbial cultures [9,10,11,12,13,14,15,16,17,18]. An HPLC method has also been used to determine this compound in *Colchicum haussknechtii* extract using a response surface methodology [19]. This compound has been analyzed in various biological fluids, such as blood serum, blood plasma, and urine samples, using an HPLC approach [11,20,21]. For its determination in serum and plasma samples, various liquid chromatography–mass spectrometry (LC–MS) methods have been reported [1,22,23,24]. For the determination of this compound in various commercial formulations and plant extracts, distinct high-performance thin-layer chromatography (HPTLC) assays have been reported [10,25,26,27,28,29,30,31,32,33,34,35]. Some electrochemical techniques have also been utilized for the determination of this compound [36,37]. The detection of this compound in plant extract has been reported using a fluorescent immunoassay [38]. A multiple-pulse amperometric technique has been reported for its analysis in pharmaceutical formulations and urine samples [39]. Its detection has also been reported using capillary electromigration techniques [40]. The range of analytical techniques for CLH detection has been shown in published studies. However, the greenness profile of the analytical techniques has not been evaluated. Furthermore, no CLH analysis has been recorded using greener HPTLC techniques. Various quantitative analytical methodologies have been utilized in the literature to assess the greenness score [41,42,43,44,45]. However, only the Analytical GREENness (AGREE) approach utilizes all 12 principles of green analytical chemistry (GAC) [43]. As a consequence, AGREE approach was used for the assessment of greenness score of the current HPTLC methods [43].

The goal of this study was to design and validate a reversed-phase HPTLC method for CLH analysis in traditional (TE) and ultrasonication-based extracts (UBE) of commercial Unani formulations, commercial allopathic formulations, and *C. autumnale* obtained from Egypt and India. This technique was then compared to the regular normal-phase HPTLC method. For the regular analytical method, routine solvent mixtures were used as the mobile phase. However, for the greener analytical method, green solvents mixtures were used as the mobile phase. Using the International Council for Harmonization (ICH) Q2-R1 guidelines, regular and greener HPTLC approaches for CLH analysis were validated [46].

## 2. Results and Discussion

### 2.1. Method Development

**Figure 1:**
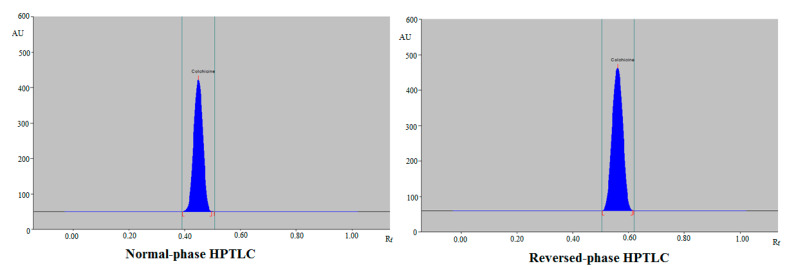
Representative chromatograms of standard colchicine (CLH) obtained using regular normal-phase high-performance thin-layer chromatography (HPTLC) and greener reversed-phase HPTLC approaches.

To establish a reliable band for CLH analysis using the regular HPTLC method, different amounts of chloroform (CHCl_3_) and methanol (MeOH), including CHCl_3_/MeOH (40:60, *v*/*v*), CHCl_3_/MeOH (50:50, *v*/*v*), CHCl_3_/MeOH (60:40, *v*/*v*), CHCl_3_/MeOH (70:30, *v*/*v*), CHCl_3_/MeOH (80:20, *v*/*v*), and CHCl_3_/MeOH (90:10, *v*/*v*), were examined as regular mobile phases. CHCl_3_/MeOH (40:60, *v*/*v*), CHCl_3_/MeOH (50:50, *v*/*v*), CHCl_3_/MeOH (60:40, *v*/*v*), CHCl_3_/MeOH (70:30, *v*/*v*), and CHCl_3_/MeOH (80:20, *v*/*v*) showed poor chromatographic peaks of CLH with high asymmetry factor (As) value (As > 1.20), whereas CHCl_3_/MeOH (90:10, *v*/*v*) presented a well-resolved and intact chromatographic peak for CLH at a retention factor (R_f_) = 0.44 ± 0.01 (Figure 1). CLH was also predicted to have As values of 1.07, which is trustworthy. As a result, CHCl_3_/MeOH (90:10, *v*/*v*) was selected as the final regular mobile phase for CLH analysis using the regular HPTLC approach.

To establish a reliable band for CLH analysis using the greener HPTLC approach, different proportions of ethanol (EtOH) and water (H_2_O), such as EtOH/H_2_O (40:60, *v*/*v*), EtOH/H_2_O (50:50, *v*/*v*), EtOH/H_2_O (60:40, *v*/*v*), EtOH/H_2_O (70:30, *v/v*), EtOH/H_2_O (80:20, *v*/*v*), and EtOH/H_2_O (90:10, *v*/*v*), were examined as greener mobile phases. EtOH/H_2_O (40:60, *v*/*v*), EtOH/H_2_O (50:50, *v*/*v*), EtOH/H_2_O (60:40, *v*/*v*), EtOH/H_2_O (80:20, *v/v*), and EtOH/H_2_O (90:10, *v*/*v*) presented poor chromatographic peak of CLH with high As value (As > 1.25), whereas EtOH/H_2_O (70:30, *v*/*v*) showed well-resolved and intact chromatographic peak of CLH at R_f_ = 0.55 ± 0.02 (Figure 1). CLH was also predicted to have As values of 1.03, which is trustworthy. As a result, EtOH/H_2_O (70:30, *v*/*v*) was selected as the final greener mobile phase for CLH analysis using the greener HPTLC approach. The greater TLC response was found at a wavelength of 354 nm for CLH when the spectral bands for CLH were examined under densitometry mode. As a consequence, the whole CLH analysis took place at 354 nm.

### 2.2. Validation Studies

Different parameters for CLH analysis were determined using the ICH-Q2-R1 guidelines [46]. Table 1 shows the results of the linear regression analysis of CLH calibration curves using both approaches. The CLH calibration curve was linear in the 100–600 ng/band range for the regular HPTLC technique and in the 25–1200 ng/band range for the greener HPTLC technique. The determination coefficient (R^2^) and regression coefficient (R) of CLH were found to be 0.9935 and 0.9967 for the regular HPTLC method and 0.9971 and 0.9985 for the greener HPTLC method, respectively. The results revealed a substantial link between CLH concentration and the measured response. All of these findings demonstrated that both approaches were suitable for CLH analysis. However, the greener HPTLC method was linear over a greater range than the regular HPTLC method.

Table 2 lists the system suitability criteria for both regular and greener HPTLC technologies. For CLH analysis, the R_f_, As, and the number of theoretical plates per meter (N/m) for the regular HPTLC approach were recorded as 0.44, 1.07, and 4464, respectively, which were satisfactory. For CLH analysis, the R_f_, As, and N/m for the greener HPTLC approach were recorded as 0.55, 1.03, and 4754, respectively, which were also satisfactory. The values of R_f_, As, and N/m for the regular and greener analytical methods were not statistically different (*p* > 0.05).

The % recovery was used to measure the accuracy of both approaches for CLH analysis. Table 3 summarizes the accuracy analysis results for both techniques. The % recovery values of CLH at three different quality-control (QC) levels were determined to be 95.41–103.09% using the regular HPTLC technique and 98.94–100.91% for the greener HPTLC methodology. Both approaches were found to be accurate for CLH analysis based on these findings. However, the % recovery of CLH using the greener analytical method was significant in terms of accuracy data compared to the regular analytical method (*p* < 0.05). Hence, for CLH analysis, the greener HPTLC method was found to be more accurate than the regular HPTLC method.

The intra/interassay precision of both approaches was investigated, and data for CLH analysis were represented as a percentage of the relative standard deviation (% RSD). Table 4 shows the results of intra/interday precisions for both techniques of CLH analysis. Tor the regular HPTLC methodology, the % RSD values of CLH for intraday and interday precision were determined to be 2.97–3.17 and 3.14–3.49%, respectively. For the greener HPTLC methodology, the % RSD values of CLH for intraday and interday precision were determined to be 0.62–0.76 and 0.60–0.84%, respectively. These data suggest that both approaches of CLH analysis were accurate, but the intraday and interday precisions of CLH using the greener analytical method were significant compared to the regular analytical method (*p* < 0.05). Hence, the greener HPTLC approach was found to be more reproducible than the regular HPTLC strategy for CLH analysis.

By introducing planned deliberate changes in the components of mobile phases, the robustness of both methods of CLH analysis was evaluated. Table 5 documents the results of robustness assessment for both methods. For the regular HPTLC method, CLH % RSD values were found to be 3.41–3.98%, while the CLH R_f_ values were determined to be 0.42–0.46. For the greener HPTLC approach, the % RSD values for CLH were determined to be 0.64–0.67%, while the R_f_ values were determined to be 0.54–0.56. These data revealed that both approaches for CLH analysis were robust. The % RSD of CLH using the greener analytical method was significant compared to the regular analytical method (*p* < 0.05). Hence, when it came to CLH analysis, the greener HPTLC method outperformed the regular HPTLC method.

The LOD and LOQ were used to assess the sensitivity of both CLH analysis methods. Table 1 shows the calculated values of LOD and LOQ for CLH using both approaches. For the regular HPTLC method, the LOD and LOQ of CLH were determined to be 34.31 ± 0.62 and 102.93 ± 1.86 ng/band, respectively. For the greener HPTLC method, the LOD and LOQ of CLH were determined to be 8.41 ± 0.10 and 25.23 ± 0.30 ng/band, respectively. These data revealed that both approaches were sensitive, but the LOD and LOQ values of CLH using the greener analytical method were significant compared to the regular analytical method (*p* < 0.05). Hence, for CLH analysis, the greener HPTLC method proved to be more sensitive than the regular HPTLC method.

### 2.3. Application of Regular and Greener HPTLC Methods in CLH Analysis in Commercial Unani Formulations, Commercial Allopathic Formulations, TE, and UBE of C. autumnale

**Figure 2:**
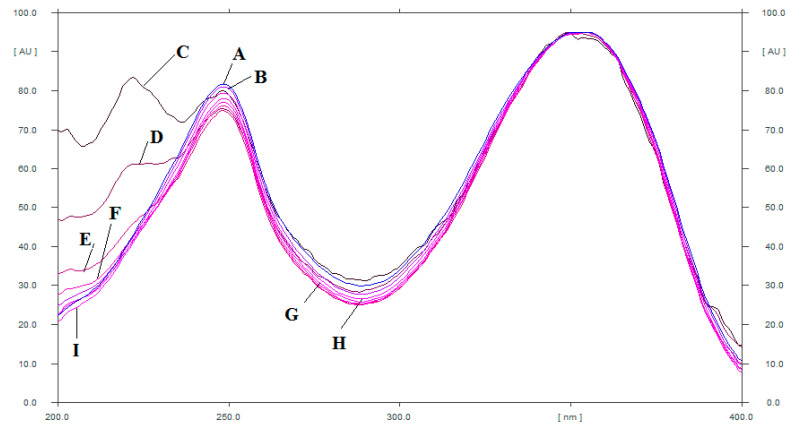
UV absorption spectra of (**A**) standard CLH, (**B**) UBE of allopathic formulation, (**C**) TE of allopathic formulation, (**D**) UBE of Indian seed extract, (**E**) TE of Indian seed extract, (**F**) UBE of Egyptian seed extract, (**G**) TE of Egyptian seed extract, and (**H**) UBE of Unani formulation, and (**I**) TE of Unani formulation superimposed.

The specificity of the proposed CLH analysis method was assessed by comparing the R_f_ values and superimposed UV absorption spectra of CLH in TE of commercial Unani formulations, UBE of commercial Unani formulations, TE of commercial allopathic formulations, TE of *C. autumnale* seed extract, and UBE of *C. autumnale* seed extract obtained from India and Egypt with those of standard CLH. The superimposed UV absorption spectra of standard CLH as well as CLH in TE of commercial Unani formulations, UBE of commercial Unani formulations, TE of commercial allopathic formulations, TE of *C. autumnale* seed extract, and UBE of *C. autumnale* seed extract obtained from India and Egypt are included in Figure 2.

At 354 nm, the greatest response of CLH in standard CLH and TE of commercial Unani formulations, UBE of commercial Unani formulations, TE of commercial allopathic formulations, TE of *C. autumnale* seed extract, and UBE of *C. autumnale* seed extract obtained from India and Egypt was determined. The specificity of the proposed HPTLC method of CLH analysis was confirmed by the identical UV absorption spectra, R_f_ values, and wavelengths of CLH in standard and TE of commercial Unani formulations, UBE of commercial Unani formulations, TE of commercial allopathic formulations, TE of *C. autumnale* seed extract, and UBE of *C. autumnale* seed extract obtained from India and Egypt.

For the determination of CLH in TE of commercial Unani formulations, UBE of commercial Unani formulations, TE of commercial allopathic formulations, TE of *C. autumnale* seed extract, and UBE of *C. autumnale* seed extract obtained from India and Egypt, both methods were applied as an alternative to traditional pharmaceutical assays. The chromatogram of CLH from Egyptian seed extract, Indian seed extract, marketed Unani tablets, and marketed allopathic tablets was verified by comparing the TLC band at R_f_ = 0.44 ± 0.01 for CLH with standard CLH using the regular HPTLC method. Figure S1 indicates the recorded chromatograms of CLH in Egyptian seed extract (Figure S1A), Indian seed extract (Figure S1B), commercial Unani formulations (Figure S1C), and commercial allopathic formulations (Figure S1D), which presented identical peak of CLH to that of standard CLH in all sample matrices studied. Some additional peaks, such as peaks 9, 8, and 7, were also found in Egyptian seed extract, Indian seed extract, and commercial Unani formulations, respectively, utilizing the regular HPTLC method. However, no extra peaks were detected in marketed allopathic tablets using the regular HPTLC methodology.

The chromatogram of CLH from Egyptian seed extract, Indian seed extract, marketed Unani tablets, and marketed allopathic tablets was verified by comparing the TLC band at R_f_ = 0.55 ± 0.02 for CLH with standard CLH using the greener HPTLC method. Figure S2 indicates the recorded chromatograms of CLH in Egyptian seed extract (Figure S2A), Indian seed extract (Figure S2B), commercial Unani formulations (Figure S1C), and commercial allopathic formulations (Figure S2D), which also presented identical peak of CLH to that of standard CLH in all sample matrices studied. Some additional peaks, such as peaks 4, 6, and 6, were also found in Egyptian seed extract, Indian seed extract, and commercial Unani formulations, respectively, utilizing the greener HPTLC approach. Using the greener HPTLC methodology, however, no extra peaks were detected in marketed allopathic tablets. The presence of additional peaks could be associated with other phytocompounds of extracts instead of CLH destruction. The presence of additional peaks indicated that both methods were suitable for CLH analysis in the presence of excipients/impurities.

**Figure 3:**
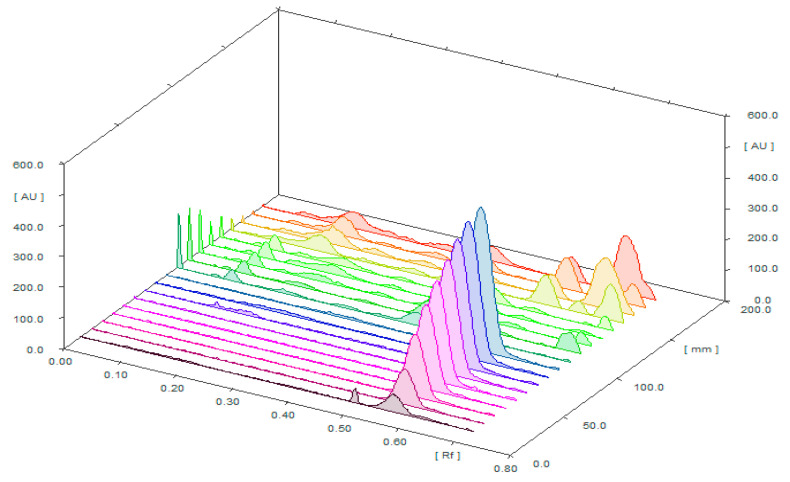
Three-dimensional TLC densitograms of standard CLH, marketed allopathic tablets, and marketed Unani tablets obtained using the greener reversed-phase HPTLC approach.

Three-dimensional TLC densitograms of standard CLH, marketed Unani formulations, and marketed allopathic tablets using the greener HPTLC method are presented in Figure 3, and they also showed similar peaks of CLH in all sample matrices. These three-dimensional densitograms indicated the selectivity and linearity of the method.

The amount of CLH was calculated using the calibration curve for the regular and greener HPTLC methods, and the results are included in Table S1. Utilizing the regular HPTLC method, the amounts of CLH in TE of Egyptian seed extract, Indian seed extract, commercial Unani formulations, and commercial allopathic formulations were determined to be 1.74 ± 0.09, 2.39 ± 0.11, 0.48 ± 0.01, and 12.45 ± 0.84% *w*/*w*, respectively. Utilizing the regular HPTLC methodology, the amounts of CLH in UBE of Egyptian seed extract, Indian seed extract, commercial Unani formulations, and commercial allopathic formulations were determined to be 1.89 ± 0.10, 2.55 ± 0.13, 0.55 ± 0.02, and 12.61 ± 0.91% *w*/*w*, respectively.

Utilizing the greener HPTLC methodology, the amounts of CLH in TE of Egyptian seed extract, Indian seed extract, commercial Unani formulations, and commercial allopathic formulations were determined to be 2.34 ± 0.12, 2.90 ± 0.14, 1.37 ± 0.04, and 13.27 ± 0.92% *w*/*w*, respectively. Utilizing the greener HPTLC methodology, the amounts of CLH in UBE of Egyptian seed extract, Indian seed extract, commercial Unani formulations, and commercial allopathic formulations were determined to be 2.61 ± 0.13, 3.19 ± 0.16, 1.58 ± 0.05, and 15.11 ± 0.97% *w*/*w*, respectively. The amount of CLH was found to be higher in all sample matrices using the greener HPTLC approach compared to the regular HPTLC approach. This observation was possible due to the use of different solvent systems in the greener and regular analytical methods. Using both approaches, the amount of CLH in the UBE of all sample matrices was higher than in the TE. As a result, the UBE procedure for CLH was deemed superior to the TE procedure. The amount of CLH in UBE of all sample matrices was not significant compared to their TE using the regular HPTLC approach. Utilizing the greener HPTLC methodology, however, the quantity of CLH in UBE of all sample matrices was considerably more than their TE. The differences in the CLH content in different extracts might be due to changes in the growth area and other environmental conditions. Overall, the greener HPTLC method was deemed superior to the regular HPTLC method for CLH pharmaceutical assay.

### 2.4. Greenness Assessment

**Figure 4:**
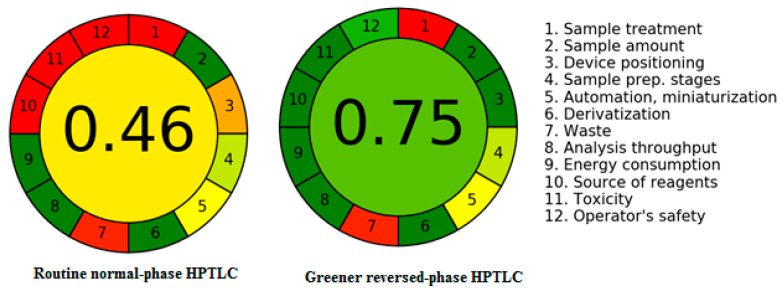
Representative pictograms for AGREE scores for the regular normal-phase HPTLC and the greener reversed-phase HPTLC methods obtained using AGREE: The Analytical Greenness Calculator.

Various methods are available for the greenness estimation of pharmaceutical assays [41,42,43,44,45]. However, only AGREE utilizes all 12 GAC principles for greenness determination [43]. As a consequence, the greenness of both methods was determined using AGREE: The Analytical Greenness Calculator (version 0.5, Gdansk University of Technology, Gdansk, Poland, 2020). Figure 4 shows a representative pictogram for the AGREE score of regular and greener HPTLC techniques. For regular and greener HPTLC procedures, the AGREE score was calculated to be 0.46 and 0.75, respectively. These data revealed that the greener HPTLC methodology had a better greenness profile than the regular HPTLC approach for CLH analysis.

## 3. Materials and Methods

### 3.1. Materials

Pure CLH was obtained from Sigma Aldrich (St. Louis, MO, USA). The chromatography-grade solvents, such as EtOH, MeOH, and CHCl_3_, were obtained from Fluka Chemica (Darmstadt, Germany). Chromatography-grade H_2_O was procured using the Milli-Q unit. The marketed Unani tablets (containing 600 µg CLH/tablet along with other excipients such as elwa, tukhm soya, turbud safaid, habb-ul-neel, suranjan shirin, gugal, and mastagi) and allopathic tablet formulations (containing 500 µg CLH/tablet along with other excipients such as lactose, pregelatinized maize starch, stearic acid, purified talc, ethanol, and purified water) of CLH were obtained from a pharmacy shop in New Delhi, India. According to the manufacturer’s guidelines, both Unani and allopathic tablets were prepared using a wet granulation technique. The composition for marketed Unani and allopathic tablets was taken from their labels. All other reagents and chemicals used were of analytical grade.

### 3.2. Plant Materials

The dried seeds of *C. autumnale* were collected from Alexandria (Egypt) and New Delhi (India). The plant materials from Alexandria (Egypt) were obtained with the geographical coordinates of 31°12′0.3312″ N and 29°55′7.4604″ E with an altitude of 5 m. The month of collection was January 2022. The plant materials from New Delhi (India) were obtained from the geographical coordinates of 28°38′41.2800″ N and 77°13′0.1956″ E with an altitude of 300 m. The month of collection was November 2021.

The identification key provided by the Saudi Arabian flora was used to verify the seeds of *C. autumnale*. The voucher specimen for *C. autumnale* (voucher number: ALX82384) was deposited in Herbarium of Department of Pharmacognosy, College of Pharmacy, Prince Sattam Bin Abdulaziz University, Al-Kharj, Saudi Arabia.

### 3.3. Instrumentation and Analytical Conditions

The HPTLC CAMAG TLC system (CAMAG, Muttenz, Switzerland) was applied for the determination of CLH in marketed Unani formulations, commercial allopathic formulations, TE, and UBE of *C. autumnale* obtained from India and Egypt. The prepared samples were applied as 6 mm bands using a CAMAG Automatic TLC Sampler 4 (ATS4) Sample Applicator (CAMAG, Geneva, Switzerland). The CAMAG microliter syringe (Hamilton, Bonaduz, Switzerland) was connected to the sample applicator. The application rate for the determination of CLH was kept constant at 150 nL/s. Under linear ascending mode, the TLC plates were established in a CAMAG automated developing chamber 2 (ADC2) (CAMAG, Muttenz, Switzerland) with a distance of 80 mm. The development chamber was saturated with vapors of respective mobile phases for 30 min at 22 °C. A wavelength of 354 nm was used to detect CLH. The slit size (band length × width) and scanning rate were set to 4 × 0.45 mm^2^ and 20 mm/s, respectively. Three or six replicates were applied for each analysis. The software utilized was WinCAT’s (version 1.4.3.6336, CAMAG, Muttenz, Switzerland).

The identical instrumentation and analytical settings were employed in both the regular normal-phase and the greener reversed-phase HPTLC techniques. The TLC plates and the mobile phase compositions showed the most significant differences between the normal-phase and reversed-phase procedures. The TLC plates were glass plates (plate size: 10 × 20 cm) precoated with normal-phase silica gel (particle size: 5 µm) 60F254S plates (E-Merck, Darmstadt, Germany) in the regular HPTLC method and RP-60F254S plates (E-Merck, Darmstadt, Germany) in the greener HPTLC method. The normal mobile phase in the regular HPTLC method was CHCl_3_/MeOH (90:10, *v*/*v*), whereas the greener mobile phase in the greener HPTLC method was EtOH/H_2_O (70:30, *v*/*v*).

### 3.4. Calibration Curves and QC Sample for CLH

CLH stock solution was made by dispensing the required amount of CLH into the given volume of mobile phase, yielding a final stock solution of 100 µg/mL CLH. CLH concentrations in the 100–600 ng/band range were obtained using the regular HPTLC method, whereas concentrations in the 25–1200 ng/band range were obtained using the greener HPTLC method, which involved diluting varying amounts of CLH stock solution with the corresponding mobile phase. An amount of 200 µL of each concentration of CLH was applied to normal-phase TLC plates for the regular HPTLC and to reversed-phase TLC plates for the greener HPTLC. The spot area of each CLH concentration was calculated using both tests. CLH calibration curves were created by plotting CLH concentrations vs. measured spot area in six replicates (n = 6). Three different QC samples were prepared freshly for the examination of different validation parameters.

### 3.5. Sample Preparation for the Determination of CLH in Commercial Allopathic and Unani Tablets Using TE

The average weight of 10 commercial allopathic tablets (each having 500 µg of CLH) or Unani tablets (each having 600 µg of CLH) was calculated. Using a glass pestle and mortar, CLH containing allopathic or Unani tablets were crushed and powdered finely. MeOH was used to extract a weight of powder equivalent to 5.0 mg of CLH. MeOH is the most effective solvent for the extraction, giving the highest extraction yields and highest content of phenolics, flavonoids, alkaloids, and terpenoids [47,48,49]. Lower temperature is needed for evaporation using MeOH extracts [47]. CLH is an alkaloidal compound and hence MeOH is the most effective solvent to obtain the highest extraction yields of CLH from *C. autumnale* [3]. Due to these reasons, MeOH was used as the solvent for the extraction of CLH. The MeOH was evaporated at 40 °C, and the residue was dissolved in 50 mL MeOH in a volumetric flask separately [47]. This technique was carried out three times. The resulting solution was utilized as a test solution for both techniques of determining CLH in the TE of commercial allopathic and Unani tablets.

### 3.6. Sample Preparation for the Determination of CLH in Marketed Allopathic and Unani Tablets Using UBE

The average weight of 10 commercial allopathic tablets (each having 500 µg of CLH) or Unani tablets (each having 600 µg of CLH) was calculated. Using a glass pestle and mortar, CLH containing allopathic or Unani tablets were crushed and powdered finely. A weight of powder equivalent to 5.0 mg of CLH was ultrasonically extracted with MeOH using Bransonic series ultrasonication vibrations (Model CPX5800H-E; Trenton, NJ, USA). A rotary vacuum evaporator was used to evaporate MeOH at 40 °C, and the residue was reconstituted with 50 mL of MeOH. The reconstituted sample was ultrasonicated at 50 °C for one hour. This technique was carried out three times. Using both procedures, the resulting solution was employed as a test solution for determining CLH in UBE of commercial allopathic and Unani tablets.

### 3.7. TE of CLH from C. autumnale Seeds Obtained from Egypt and India

The dried seeds of *C. autumnale* collected from Egypt and India were coarsely powdered. The coarsely powdered seeds (5 g) obtained from Egypt and India were extracted by maceration with MeOH (3 × 100 mL) at room temperature. Each sample was filtered using a Whatman filter paper (No. 41). The obtained seed extract of C. *autumnale* obtained from Egypt and India was evaporated separately at 40 °C under reduced pressure utilizing a rotary vacuum evaporator. Then, the concentrated extracts of different geographical regions were reconstituted with 50 mL of MeOH [48]. This procedure was carried out in triplicates. The obtained solution was used as a test solution for the determination of CLH in TE of *C. autumnale* obtained from Egypt and India using both methods.

### 3.8. UBE of CLH from C. autumnale Seeds Obtained from Egypt and India

The dried seeds of *C. autumnale* obtained from Egypt and India were ultrasonically extracted utilizing Bransonic series ultrasonication vibrations (Model CPX5800H-E; Trenton, NJ, USA). Then, 5 g of powdered dried seeds of *C. autumnale* was carefully weighed and extracted with 100 mL of MeOH. A rotary vacuum evaporator was used to evaporate MOH at 40 °C, and the residue was reconstituted with 50 mL of MeOH. The reconstituted sample was ultrasonicated at 50 °C for one hour [48]. This procedure was carried out in triplicates. The obtained solution was used as a test solution for the determination of CLH in UBE of *C. autumnale* obtained from Egypt and India using both methods.

### 3.9. Validation Studies

Regular and greener HPTLC techniques for CLH analysis were validated for several parameters using the ICH-Q2-R1 recommendations [46]. CLH linearity was determined by plotting CLH concentrations against the measured peak area. The linearity was tested in the 100–600 ng/band range (n = 6) for the regular HPTLC technique and in the 25–1200 ng/band range (n = 6) for the greener HPTLC approach.

The evaluation of R_f_, As, and N/m was used to determine the parameters for system suitability for regular and greener HPTLC approaches for the determination of CLH. For both methods, the R_f_, As, and N/m data were calculated using their reported formulae [45].

The accuracy of regular and greener HPTLC procedures for determining CLH was assessed using the percent recovery method. The accuracy for both methods was determined using CLH standard solution instead of plant extracts by the spiking method. CLH was tested at three QC levels of standard CLH solution, namely low QC (LQC; 100 ng/band), middle QC (MQC; 400 ng/band), and high QC (HQC; 600 ng/band), to determine the accuracy of the regular HPTLC approach. CLH was also tested at three different QC levels of standard CLH solution, namely LQC (50 ng/band), MQC (400 ng/band), and HQC (1200 ng/band), to determine the accuracy of the greener HPTLC approach. The percent recovery of CLH was calculated using both methods at each QC level (n = 6).

For CLH, the intra/interassay precision of regular and greener HPTLC techniques was investigated. The intraassay precision for CLH was evaluated using estimation of freshly generated CLH solutions at LQC, MQC, and HQC on the same day for both procedures (n = 6). The interassay variance was investigated using estimation of freshly generated CLH solutions at LQC, MQC, and HQC on three different days for both methods (n = 6).

The robustness of CLH was tested for both techniques by making some planned adjustments to the mobile phase components. The regular mobile phase CHCl_3_/MeOH (90:10, *v*/*v*) for CLH was modified to EtOH/H_2_O (92:8, *v*/*v*) and EtOH/H_2_O (88:12, *v*/*v*) for the regular HPTLC approach, and the variations in measured response and R_f_ values were examined (n = 6). The greener mobile phase EtOH/H_2_O (70:30, *v*/*v*) for CLH was modified to EtOH/H_2_O (72:28, *v*/*v*) and EtOH/H_2_O (68:32, *v*/*v*) for the greener HPTLC approach, and the variations in measured response and R_f_ values were examined (n = 6).

Using a standard deviation approach, the sensitivity of regular and greener HPTLC techniques for CLH was determined as limit of detection (LOD) and limit of quantification (LOQ). The LOD and LOQ were calculated using their published formulae for both methods (n = 6) [46].

The R_f_ values and UV absorption spectra of CLH in marketed Unani formulations, commercial allopathic formulations, TE, and UBE of *C. autumnale* obtained from India and Egypt were compared to those of standard CLH to investigate the specificity of regular and greener HPTLC approaches for CLH.

### 3.10. Application of Regular and Greener HPTLC Methods in the Determination of CLH in Commercial Unani Formulations, Commercial Allopathic Formulations, TE, and UBE of C. autumnale

The processed samples of marketed Unani formulations, commercial allopathic formulations, TE, and UBE of *C. autumnale* obtained from India and Egypt were spotted on normal-phase TLC plates for the regular HPTLC method and on reversed-phase TLC plates for the greener HPTLC approach. For both methods, the chromatographic responses were examined utilizing the same experimental conditions used for the determination of CLH (n = 3). For both methods, the quantity of CLH in commercial Unani formulations, commercial allopathic formulations, TE, and UBE of *C. autumnale* obtained from India and Egypt were estimated using the calibration curve of CLH.

### 3.11. Greenness Estimation

The AGREE approach [43] was used to examine the greenness profile for regular and greener HPTLC procedures for CLH analysis. The AGREE scores (0.0–1.0) for regular and greener reversed-phase HPTLC methods was obtained using AGREE: The Analytical Greenness Calculator (version 0.5, Gdansk University of Technology, Gdansk, Poland, 2020) for both methods.

### 3.12. Statistical Evaluation

The validation parameters of regular and greener HPTLC approaches were analyzed and compared using Student’s *t*-test utilizing the MS Excel 2010 program. The *p*-value of less than 0.05 was considered as significant.

## 4. Conclusions

For CLH analysis, there is a paucity of greener analytical approaches in the literature. Consequently, this research attempted to develop and validate a rapid, sensitive, and greener HPTLC method for CLH analysis in diverse commercial formulations and plant extracts and then compare it to the regular HPTLC methodology. The greener HPTLC methodology was found to be more linear, accurate, precise, robust, and sensitive for CLH analysis than the regular HPTLC method. When comparing the greener HPTLC technique to the regular HPTLC approach, the amount of CLH in all sample matrices was found to be significantly higher in terms of % *w*/*w*. The AGREE results showed that the greener HPTLC approach had a better greenness profile than the regular HPTLC method. Overall, based on numerous validation criteria and pharmaceutical assay findings, the greener HPTLC methodology is superior to the regular HPTLC method for CLH analysis in commercial formulations and plant extracts. These results suggest that the greener analytical methodology can be used for the analysis of CLH in a wide range of sample matrices.

## Figures and Tables

**Table 1:** Results for the regression analysis of colchicine (CLH) for the regular normal-phase high-performance thin-layer chromatography (HPTLC) and the greener reversed-phase HPTLC approaches (mean ± SD; n = 6).

Parameters	Normal-Phase HPTLC	Reversed-Phase HPTLC
Linearity range (ng/band)	100–600	25–1200
Regression equation	y = 15.039x + 1497.9	y = 20.837x + 800.37
R^2^	0.9935	0.9971
R	0.9967	0.9985
Standard error of slope	0.38	0.40
Standard error of intercept	13.57	3.03
95% confidence interval of slope	13.38–16.69	19.11–22.55
95% confidence interval of intercept	1439.49–1556.30	787.29–813.44
LOD ± SD (ng/band)	34.31 ± 0.62	8.41 ± 0.10
LOQ ± SD (ng/band)	102.93 ± 1.86	25.23 ± 0.30

R^2^: determination coefficient; R: regression coefficient; LOD: limit of detection; LOQ: limit of quantification.

**Table 2:** System suitability parameters of CLH for the regular normal-phase HPTLC and the greener reversed-phase HPTLC approaches (mean ± SD; n = 3).

Parameters	Normal-Phase HPTLC	Reversed-Phase HPTLC
R_f_	0.44 ± 0.01	0.55 ± 0.02
As	1.07 ± 0.02	1.03 ± 0.01
N/m	4464 ± 3.74	4754 ± 3.91

R_f_: retention factor, As: asymmetry factor, N/m: number of theoretical plates per meter.

**Table 3:** Assessment of accuracy of CLH for the regular normal-phase HPTLC and the greener reversed-phase HPTLC approaches (mean ± SD; n = 6).

Conc. (ng/band)	Conc. Found (ng/band) ± SD	Recovery (%)	RSD (%)
	Normal-Phase HPTLC		
100	95.41 ± 3.02	95.41	3.16
400	387.32 ± 11.23	96.83	2.89
600	618.54 ± 15.24	103.09	2.46
	Reversed-phase HPTLC		
50	50.12 ± 0.41	100.24	0.81
400	403.65 ± 2.85	100.91	0.70
1200	1187.32 ± 7.45	98.94	0.62

**Table 4:** Assessment of intra/interday precision of CLH for the regular normal-phase HPTLC and the greener reversed-phase HPTLC approaches (mean ± SD; n = 6).

Conc. (ng/band)	Intraday Precision			Interday Precision		
	Conc. Found *(ng/band)* ± SD	Standard Error	RSD (%)	Conc. Found *(ng/band)* ± SD	Standard Error	RSD (%)
Normal-phase HPTLC						
100	103.21 ± 3.28	1.33	3.17	104.24 ± 3.64	1.48	3.49
400	406.85 ± 12.34	5.03	3.03	407.84 ± 13.21	5.39	3.23
600	584.32 ± 17.41	7.10	2.97	597.23 ± 18.24	7.44	3.14
Reversed-phase HPTLC						
50	49.87 ± 0.38	0.15	0.76	49.63 ± 0.42	0.17	0.84
400	397.56 ± 2.59	1.05	0.65	405.61 ± 2.61	1.06	0.64
1200	1212.31 ± 7.52	3.07	0.62	1184.32 ± 7.21	2.94	0.60

**Table 5:** Assessment of robustness for the regular normal-phase HPTLC and the greener reversed-phase HPTLC approaches (mean ± SD; n = 6).

Conc. (ng/band)	Mobile Phase Composition (Chloroform-Methanol)			Results		
	Original	Used		Conc. *(ng/band)* ± SD	RSD (%)	R_f_
Normal-phase HPTLC						
		92:8	+2.0	386.32 ± 13.21	3.41	0.42
400	90:10	90:10	0.0	396.94 ± 14.32	3.61	0.44
		88:12	−2.0	407.51 ± 16.24	3.98	0.46
Reversed-phase HPTLC						
Mobile phase composition (ethanol-water)						
		72:28	+2.0	392.41 ± 2.54	0.64	0.54
400	70:30	70:30	0.0	402.12 ± 2.67	0.66	0.55
		68:32	−2.0	406.32 ± 2.76	0.67	0.56

## Data Availability

Not applicable.

## Supplementary Materials

The following supporting information can be downloaded at: https://www.mdpi.com/article/10.3390/plants11131767/s1. Figure S1: Representative chromatograms of CLH in (A) Egyptian seed extract, (B) Indian seed extract, (C) Unani formulation, and (D) allopathic formulation obtained using regular normal-phase HPTLC approach; Figure S2: Representative chromatograms of CLH in (A) Egyptian seed extract, (B) Indian seed extract, (C) Unani formulation, and (D) allopathic formulation obtained using greener reversed-phase HPTLC approach; Table S1: Application of the regular normal-phase HPTLC and the greener reversed-phase HPTLC approaches in the determination of CLH in methanolic extracts of Egyptian and Indian *C. autumnale* seeds, allopathic formulation, and Unani formulation produced by traditional and ultrasonication procedures (mean *±* SD; n = 3).
