# Supplementary material for: Determination of Colchicine in Pharmaceutical Formulations, Traditional Extracts, and Ultrasonication-Based Extracts of Colchicum autumnale Pleniflorum (L.) Using Regular and Greener HPTLC Approaches: A Comparative Evaluation of Validation Parameters
Source: Plants (Basel). 2022 Jul 3;11(13):1767. doi: 10.3390/plants11131767 (PMC9268838; doi:10.3390/plants11131767)
Supplement: Supplementary file 1 [file plants-11-01767-s001.zip › plants-1796453-supplementary.pdf]

Supplementary Materials

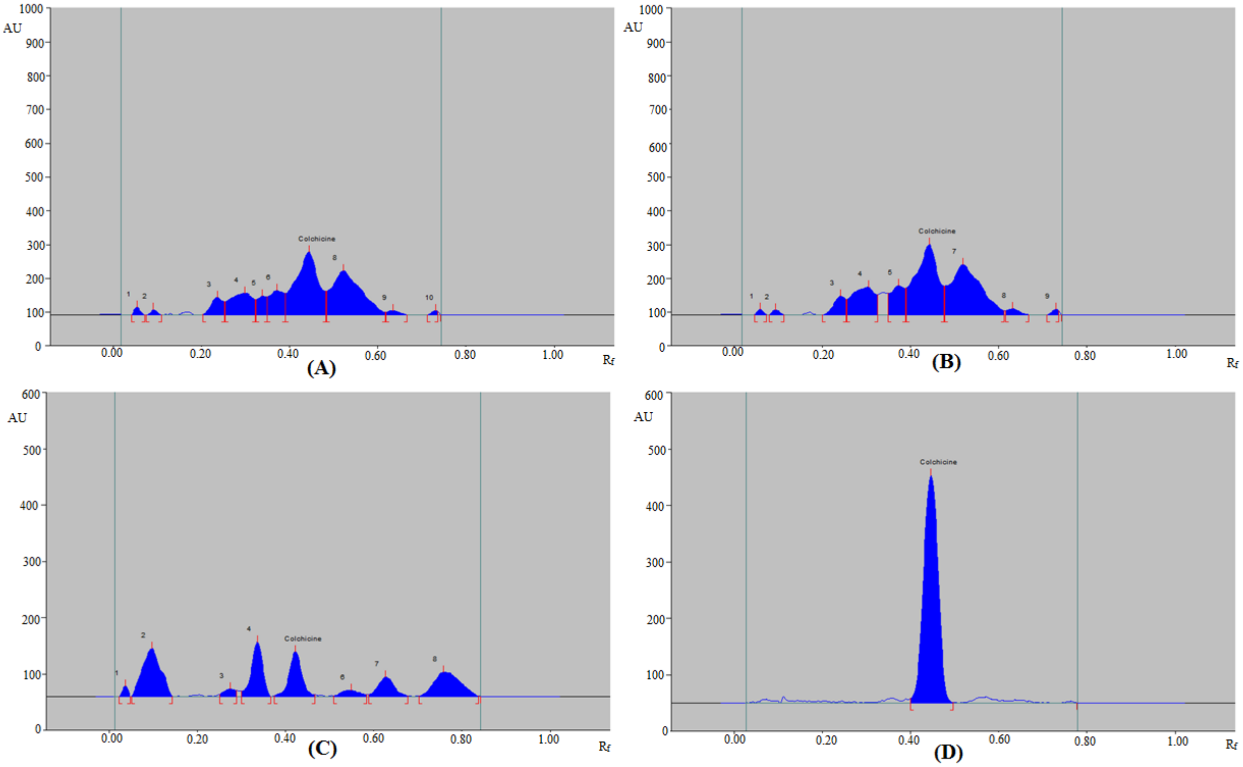

Figure S1. Representative chromatograms of CLH in (A) Egyptian seed extract, (B) Indian seed extract, (C) Unani formulation, and (D) allopathic formulation obtained using regular normal-phase HPTLC approach.

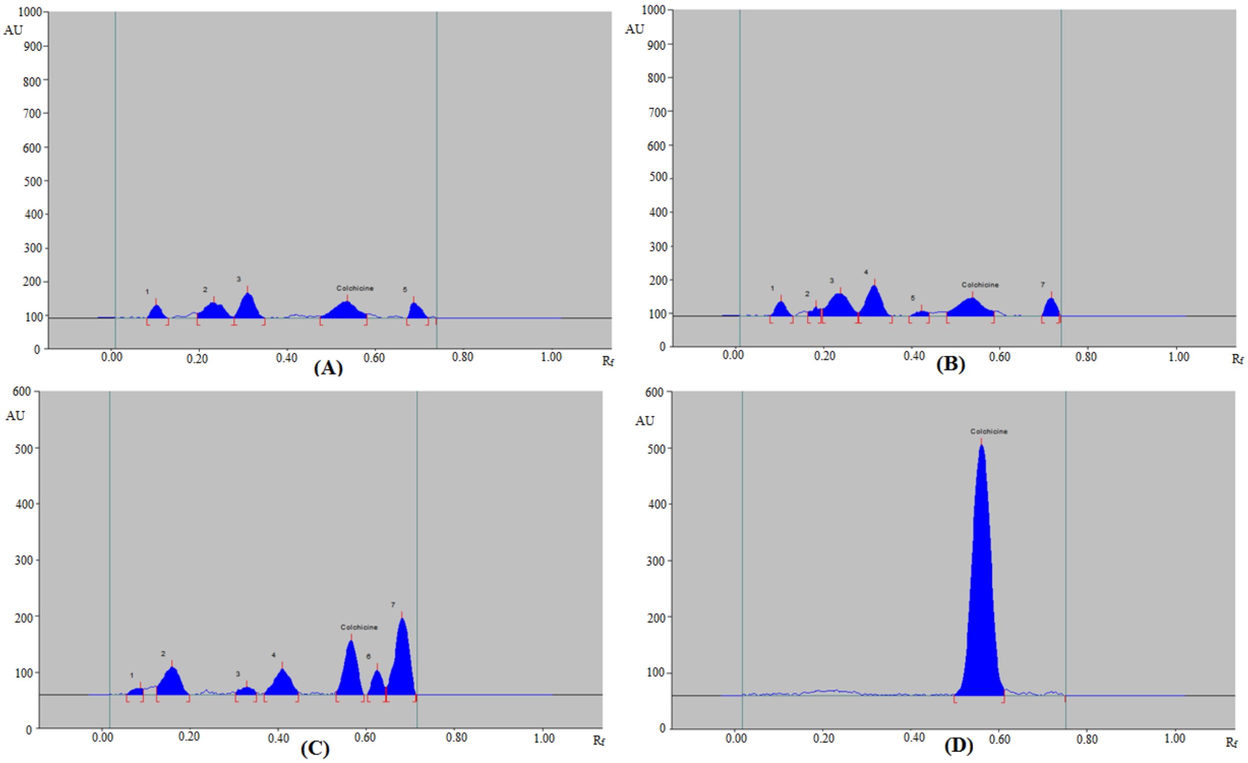

Figure S2. Representative chromatograms of CLH in (A) Egyptian seed extract, (B) Indian seed extract, (C) Unani formulation, and (D) allopathic formulation obtained using greener reversed-phase HPTLC approach.

Table S1. Application of the regular normal-phase HPTLC and the greener reversed-phase HPTLC approaches in the determination of CLH in methanolic extracts of Egyptian and Indian *C. autumnale* seeds, allopathic formulation, and Unani formulation produced by traditional and ultrasonication procedures (mean ± SD; n = 3).

Samples	Traditional extraction	Ultrasonication-based extraction
**Amount of CLH (% *w*/*w*) using normal-phase HPTLC**		
C. autumnale (Egyptian)	1.74 ± 0.09	1.89 ± 0.10
C. autumnale (Indian)	2.39 ± 0.11	2.55 ± 0.13
Allopathic formulation	12.45 ± 0.84	12.61 ± 0.91
Unani formulation	0.48 ± 0.01	0.55 ± 0.02
**Amount of CLH (% *w*/*w*) using reversed-phase HPTLC**		
C. autumnale (Egyptian)	2.34 ± 0.12	2.61 ± 0.13
C. autumnale (Indian)	2.90 ± 0.14	3.19 ± 0.16
Allopathic formulation	13.27 ± 0.92	15.11 ± 0.97
Unani formulation	1.37 ± 0.04	1.58 ± 0.05
